# JIVR: a new frontier for global injury and violence prevention

**DOI:** 10.5249/jivr.v1i1.79

**Published:** 2009-07

**Authors:** Babak Izadi, Mahmoudreza Moradi, Alireza Ahmadi, Shahrzad Bazargan-Hejazi, Behrooz Hamzeh, Omid Beiki, Davoud Khorasani-Zavareh, Hassan Haghparast, Homayoun Sadeghi-Bazargani, Farid Najafi

**Affiliations:** ^*a*^Kermanshah University of Medical Sciences, Iran.; ^*b*^Karolinska Institute, Stockholm, Sweden.; ^*c*^Charles Drew University/UCLA, United States.

Welcome to the JIVR! On behalf of Editorial Team/Board, we are delighted to inform you that after three years of preparation and hard work the journal entitled "Journal of Injury and Violence Research" (JIVR) is established. The driving force behind the inception and evolution of JIVR has been to provide an interdisciplinary forum for publishing definitive peer reviewed articles devoted to the clinical practice of Injury and Violence. The JIVR aims be a participant in continuing education of clinicians, forecast important issues and trends in Injury and Violence prevention (such as Trauma, anti-violence against self and others, sport safety, road safety, child safety, disaster control and all intention and unintentional injuries research) and foster responsible debate on injury and violence related controversial issues. As an international journal, it welcomes cross-communication between different cultures and encourages interdisciplinary discussions in the field of injury and violence prevention and treatment.

It is with a great pride and joy that we are starting to write this editorial message for the inaugural issue of the Journal. The past three years of preparation have been filled with defiantly hard efforts, exhilaration and some anxiety. We are grateful to all the authors, reviewers, and editors who have been supporting this journal during its infancy. We are equally excited to see the fruits of our labors and indeed, as with all other new journals, JIVR is commencing the print of its 1st issue.

The journal’s long-term goals are as follow:

1. To produce scholarly publications in a user-friendly medium so that our injury and violent prevention colleagues around the globe will be determine to read and enthusiastic to publish in JIVR.
2. To index JIVR in the PubMed/MEDLINE during the 2nd year of publication (once indexed, all articles starting from the issue 1 will be retroactively cited).
3. To obtain an Impact Factor (IF) when eligible, and continually strive toward improving it.
4. To encourage injury and violence prevention outcomes research in order to formally demonstrate our value to the key stakeholders.
5. To become the “go to” injury and violence prevention journal for transmitting state-of-the-art information.

In conclusion, we want to thank our readers and all the other constituents involved in this effort for recognizing JIVR, much has been accomplished and achieved as a result. However, the transitional nature of any new journal invites challenges and barriers. With your support and the endless efforts of the entire editorial board and the staff, and JIVR commitment to producing high-quality publication, these challenges will be overcome. We have seen the “Blue, Red and White” journals; now welcome to “The Green Journal”! A color of peace-loving, nonviolent, calm, tranquil, injury-free, and also healthy and balance living (after all, “Green” derives from the mixture of Yellow and Blue). The Green Journal has arrived. We urge you to embrace it as it is your journal and we look forward to your ongoing feedback.

